# Organizational preparedness for the use of large language models in pathology informatics

**DOI:** 10.1016/j.jpi.2023.100338

**Published:** 2023-10-01

**Authors:** Steven N. Hart, Noah G. Hoffman, Peter Gershkovich, Chancey Christenson, David S. McClintock, Lauren J. Miller, Ronald Jackups, Vahid Azimi, Nicholas Spies, Victor Brodsky

**Affiliations:** a Division of Computational Pathology and AI, Department of Laboratory Medicine and Pathology, Mayo Clinic, Rochester, MN, United States; b Department of Laboratory Medicine and Pathology, University of Washington, Seattle, WA, United States; c Yale Medical School Department of Pathology, New Haven, CT, United States; d Department of Laboratory Medicine and Pathology, Mayo Clinic, Jacksonville, FL, United States; e Department of Pathology, University of Michigan, Ann Arbor, MI, United States; f Department of Pathology and Immunology, Washington University School of Medicine, St. Louis, MO, United States

**Keywords:** Large language models, Artificial intelligence, Best practices

## Abstract

In this paper, we consider the current and potential role of the latest generation of Large Language Models (LLMs) in medical informatics, particularly within the realms of clinical and anatomic pathology. We aim to provide a thorough understanding of the considerations that arise when employing LLMs in healthcare settings, such as determining appropriate use cases and evaluating the advantages and limitations of these models.

Furthermore, this paper will consider the infrastructural and organizational requirements necessary for the successful implementation and utilization of LLMs in healthcare environments. We will discuss the importance of addressing education, security, bias, and privacy concerns associated with LLMs in clinical informatics, as well as the need for a robust framework to overcome regulatory, compliance, and legal challenges.

## Introduction

### What are Large Language Models (LLMs) and why are they a big deal?

Large Language Models (LLMs) represent a revolutionary category of artificial intelligence (AI) systems. Unlike their predecessors, LLMs distinguish themselves through their vast scale and the ability to learn from a wide array of data sources. These models are trained on extensive datasets of text via an unsupervised learning process, and subsequently fine-tuned for specific tasks. This enables them to discern patterns within text autonomously, thereby allowing them to consider context, generate coherent sentences (typically in response to a prompt), and execute various tasks with greater accuracy than earlier systems.

The year 2022 saw the rise of the ChatGPT web app,^1^ which utilized the GPT-3.5 model, bringing LLMs into the limelight with its fluent, rapid, and insightful responses in multiple languages. This surge has since sparked a flurry of competing products (Table 1) and associated tools.^2^ However, this list is only a glimpse into some of the more well-known models, is far from exhaustive and is continuously evolving (see Yang et al.^3^ and Hugging Face^4^ for a more comprehensive list).

**Table 1:** Abbreviated list of LLMs.

LLM title	License	Distinguishing features
PaLM^65^	Closed source	Efficient hardware utilization
Med-PaLM 2^7^	Closed source	Trained on medical knowledge
ChatGPT^1^	Closed source	Popular, freely accessible
GPT-4^66^	Closed source	Accepts and interprets images
LLaMa^67^	Closed source (leaked online)	Fine-tunable weights
RedPajama^68^	Apache License 2.0	Clean-room implementation of LLaMa
Alpaca^69^	CC BY NC 4.0	Enables affordable retraining
RoBERTa^70^	MIT	An optimized BERT technique
ColossalAI^71^	Apache License 2.0	Collection of parallel components
OpenLLaMa^9^	MIT	An open reproduction of LLaMa
StableLM^72^	CC BY-SA-4.0, CC BY-NC-SA-4.0, Apache License 2.0	From the makers of Stable Diffusion
HuggingChat^73^	Apache License 2.0	From Hugging Face
ReplitLM^74^	CC BY-SA-4.0	A source code development tool
StarCoder^75^	OpenRAIL & Apache License 2.0	A source code development tool
ImageBind^76^	CC-BY-NC 4.0	Multisensory/multi-modal
MLC LLM^77^	Apache License 2.0	Aims to run locally on phones
Web LLM^78^	Apache License 2.0	Runs in browser on client side
LLaVa^79^	Apache License 2.0, CC BY NC 4.0	Works with Image data
InstructGPT^80^	Closed source	Fine-tuned with human feedback
MPT-7B^81^	Apache License 2.0	Takes long input; commercial use
Anthropic Claude^70^	Closed source	Accepts up to 100K tokens

A distinction should be made between LLMs and the conversational interfaces used to interact with them, often called “chatbots”. A chatbot is any application that uses natural language processing techniques to simulate human conversation. These chatbots vary with respect to the complexity of the models, with some relying on simple rule-sets or “if-then” statements, to complex applications like ChatGPT built on top of state-of-the-art LLMs such as GPT-3.5 or GPT-4.

The evolution of LLMs is occurring at a staggering pace. Recent advancements have enabled these models to enhance their own accuracy through self-reflection on their errors.^5^ Notably, ChatGPT has even passed the Step 1, Step 2CK, and Step 3 exams from the United States Medical Licensing Examination (USMLE),^6^ underscoring its potential for practical applications. Med-PaLM 2 achieved an 85% score on the MedQA dataset of USMLE-style questions and was the first to pass the MedMCQA set of Indian AIIMS and NEET medical examination questions with a score of 72.3%.^7^

Considering these advancements, an increasing number of organizations have shown interest in developing LLMs for use in their own practice. However, the computational costs associated with training LLMs “from scratch” are substantial, often running into hundreds of thousands of dollars and requiring enormous computational resources.^8^ Nevertheless, organizations need not construct their own LLMs to reap the benefits—utilizing or fine-tuning existing trained LLMs is already a more affordable and accessible option for most.

The potential influence of LLMs on the global job market is immense, with the most extreme estimates suggesting an impact on approximately 300 million jobs across major economies.^9^ The popularity of LLMs was vividly demonstrated when the ChatGPT app set a record for the fastest growing user base of any online application, amassing 100 million users within 2 months of its November 2022 release.^10^ Given the public's growing interest in LLMs, it is unsurprising that various industries are recognizing the potential of this technology, as well as the risk of being left behind if they fail to adopt it. The healthcare sector, including laboratory medicine and pathology, is no exception. However, the ways in which this technology will be utilized, and the necessary preparations, are still largely undetermined. Therefore, it is crucial for informaticists, pathologists, and laboratorians to understand the capabilities and implications of LLMs. This understanding will serve as the first step in establishing organizational readiness to harness this technology.

### How do LLMs work?

The fundamental unit of LLM inputs and outputs is a “token”, which represents the linguistic “atoms” of text. These tokens can be individual characters, punctuation marks, words, or even entire phrases derived from the initial text processing or “tokenization”. The ultimate objective of an LLM is to predict the most plausible sequence of subsequent tokens based on any given set of input tokens. The full set of input tokens, or “context window” is combined with the model’s pretrained weights to generate a semantic representation of the inputs (the “embeddings”) which is then processed through the model. When iteratively interacting with a model, such as in a conversation, the context window includes the most recent prompt plus the inputs and outputs of that which came before it. This leads to a behavior wherein it seems like the models “remember” the earlier parts of the conversation. As input token sizes continue to increase, this “memory” will improve accordingly.

An additional layer of training known as “reinforcement learning with human feedback” (RLHF) [XXX cite Ouyang] has proven to be a key component of the most performant models: if an output that aligns well with a human observer's expectations, then the model is rewarded, prompting the LLM to update its policy to generate similar future outputs. LLMs can also be “fine-tuned” to produce outputs that are more specific to a particular topic, style, or use-case. This process involves exposing a trained LLM to a much narrower body of knowledge, while providing labels or feedback that guides the outputs towards the desired style.

Until recently, LLMs were trained, tuned, and deployed as static entities, with their “knowledge” of the world ceasing at their most recent training cycle. However, this paradigm has been revolutionized by OpenAI's introduction of plug-ins for its ChatGPT service. By granting these models access to external resources, they can generate responses on current events, interact with webpages, public datasets, and much more. New tools using this strategy will significantly lower the barrier to implementing, fine-tuning, and deploying customized LLMs within an organization.

While the primary function of current LLMs is to respond to text queries with “predicted” text, other transformer-based or related algorithms have been recently used to successfully generate images, videos, sounds, or other data in response to text (and vice versa). Notable examples of these efforts include GPT-4,^11^ which accepts image inputs, AudioGPT^12^ for audio inputs, and Stable Diffusion^13^ to convert text prompts to images. These approaches can further benefit from incorporating ControlNets^14^ into the architecture to enable the input of additional crucial discrete data that defines the nature of the output.

The increased accessibility of LLMs, coupled with the reduced barriers to their implementation, has profound implications for their adoption and utility across various industries. By lowering the barriers to entry, these tools can democratize the use of LLMs, enabling more organizations and individuals to leverage their potential for innovation and problem-solving. However, as with most advancements, the potential they offer comes with a significant responsibility. This necessitates further investigation into the appropriate and responsible use of LLMs, as well as increased regulatory oversight across the multiple disciplines affected, including healthcare.^15^, ^16^, ^17^

## Literature describing LLMs in pathology informatics

While the body of literature is not extensive, several publications have begun to shed light on the potential applications of LLMs in the realm of pathology informatics. For example, studies have demonstrated their efficacy in tasks such as concept extraction, medical relation extraction, semantic textual similarity, natural language inference, and medical question answering from Electronic Health Record (EHR) data.^18^ These applications hold the potential to enhance diagnostic precision, identify patterns, and optimize patient treatment plans. Additional research has delved into the use of LLMs for generating pathology reports^19^ and automating the coding of diagnoses,^20^ providing pertinent textual descriptions and diagnoses,^21^ and classifying whole slide images.^22^ These advancements could help alleviate manual workloads and boost the efficiency of healthcare professionals. However, LLMs can struggle to identify relevant information—particularly in long-text inputs,^23^ and as of yet there are no large-scale, independent, or rigorous evaluations on directly applicable pathology informatics tooling for use in clinics.

## Potential use cases for LLMs in pathology informatics

Large Language Models hold a plethora of potential applications in healthcare, many of which are directly applicable to pathology informatics.^24^ In this section, we briefly explore theoretical use cases spanning data processing and extraction, report generation and summarization, coding and billing, diagnostic support, quality control, and education and training.

### Data processing and extraction

LLMs can discretize data from pathology notes, identify coded concepts corresponding to free text and/or discrete input data, and extract data from tabular data using natural language prompts. They can also generate SQL queries given a database schema and source code based on a textual description. Furthermore, LLMs can recommend data analysis strategies based on the provided discrete data set and construct the corresponding data visualizations.^25^

### Support and communication

With appropriate preparation and prompting, LLMs could potentially serve a front-desk support role, triaging requests to the informatics group, recommending next steps, and even submitting relevant requests via other support ticket systems. Similarly, a validated LLM could be tasked with communicating basic concepts of pathology report contents to a patient via the patient portal, explaining the diagnosis in layman's terms and answering questions about it.

### Report generation and summarization

LLMs can be employed to summarize case visits, documents, and manuscripts,^26^ as well as rephrase documents and create slideshow presentations from supplied documents.^27^ In the realm of coding and billing, LLMs can identify billing/CPT codes and ICD10 codes, thereby streamlining the administrative aspect of pathology informatics and optimizing billing processes.

### Patient accessibility

LLMs can be used to provide more accessible presentations of the information within medical results and reports. Given that the reading comprehension of the average patient enrolled in Medicare is estimated at that of a fifth-grader,^28^ an application that can translate our reports into more digestible language may help reduce disparities and improve access to follow-up care. GPT-4 demonstrated such ability on radiology reports in a recent pre-print article,^29^ and a properly validated LLM could even be tasked with answering patient’s questions about the report interactively.

### Diagnostic support

An appropriately validated LLM might provide diagnostic support by generating lists of differential diagnoses, suggesting additional or alternative laboratory test orders based on EHR chart contents, recommending special stains, initiating reflex testing, and offering anomaly detection. For quality control, they can perform EHR chart-based QC, case-finding, and study cohort selection. Interestingly, LLMs can also accelerate the training of AI recognition models by potentially generating dataset labels more efficiently than humans.^30^^,^^31^

### Education and training

LLMs can generate step-by-step solutions to problems with free-text explanations, residency or fellowship application essays, cover letters, draft abstracts, papers, textbook chapters, and grant applications. They can also be employed to review grant applications, conduct quality assurance of pathology reports, summarize and preliminarily rank residency, fellowship, and job applications, as well as generate exam and quiz questions. Although the aforementioned use cases may neither be appropriate nor wise, the potential (and perhaps inevitable) use of LLMs for these activities highlights the need for carefully considered policies and controls to avoid introducing errors and bias into processes that can shape careers.

It is important to note that this list is far from exhaustive and merely scratches the surface of what is already possible using today's technology. The use of tomorrow's technology is difficult to imagine, but given the interest, adoption, and overall impressiveness of these new AI models, the capabilities of pathology informatics are bound to expand, making it an increasingly attractive field for current and future pathologists.

## When NOT to use LLMs in pathology informatics

In the realm of pathology informatics, the suitability of employing generative AI models such as LLMs hinges on the specific context and potential risks associated with the application. While LLMs can offer substantial benefits, there are circumstances where their use may be inappropriate or even detrimental.

One useful framework for evaluating the suitability of an LLM for a given use case is to consider whether the output of a request can be directly evaluated based on the content of the inputs (e.g., summarization or feature extraction), or whether the response depends more explicitly on the validity of information embedded in the model (e.g., rendering a diagnosis from a case history or identifying citations for a research topic). For example, most would concur that it would be ill-advised to depend on the current generation of LLMs for unsupervised primary diagnostic opinions when an experienced human provider is available and has the time to make the diagnosis. Conversely, the use of LLMs for secondary diagnosis and/or secondary review of clinical data can be both beneficial and more widely accepted, as demonstrated by a case where ChatGPT reportedly saved a dog's life,^32^ or one in which a child was finally diagnosed after many years of chronic pain.^33^ The risk of incorrect diagnoses and subsequent adverse outcomes from the use of AI should always be carefully considered, although in some cases, AI can be used as an aid in the process.^34^ For example, an LLM that converts a natural language translation to a SQL query can be validated by an expert before any potential harm occurs. LLMs should not be used when results cannot be independently verified, and verification by another independent LLM instance will likely not be sufficient, as the same biases or errors might be present in both models.

Notable failures of LLMs^35^^,^^36^ serve as a reminder that these models should be used with caution, particularly in high-stake situations. Currently available LLMs struggle to provide opinions on controversial topics in a fair and balanced manner, likely due to biases in the training set or the absence of universally objective definitions of bias in their output. This issue has been underscored in the context of mental health chatbots.^37^ Service eligibility determination, such as in insurance, may also be an inappropriate use of LLMs due to biases and lack of explainability. In some instances, LLMs may even be prohibited by government institutions,^38^ so their use in such constrained environments is not recommended. Moreover, many journals and websites now have policies against naming LLMs as authors in scientific publications, indicating that their use in authorship may be considered inappropriate.

While this section's goal was to outline when not to use LLMs in pathology informatics, it's important to note that there are tasks for which these models are particularly well-suited, including those listed in the previous section. Many operationally focused use cases that are amenable to human supervision are already well within the capabilities of current models. Overall, the early use of LLMs in pathology and clinical laboratories will be most beneficial when their outputs can be verified and validated by human experts. A careful consideration of the context, risks, and potential benefits is crucial when deciding whether to use LLMs in pathology informatics.

## Preparing your organization for life with LLMs

Given the breadth of potential use cases and the novelty of the technology, each organization can expect that the process for introducing LLMs will include a great deal of trial and error and discovery. The requirements for each organization will depend heavily on the intended use cases, so it is critical to consider specific objectives early in the process. One can predict that the advent of LLMs in pathology informatics will need to be accompanied by a comprehensive approach to education and training across various roles within the healthcare ecosystem. Embracing LLMs as an essential tool, akin to the introduction of healthcare calculators or nomograms in the past, requires a thorough understanding of their potential and limitations among different stakeholders.

Pathologists will need to be made aware of the capabilities and limitations of specific instances of LLM-enabled products to effectively incorporate these models into their diagnostic and reporting workflows. Laboratory technologists and information technology (IT) analysts should also be educated to the capabilities and potential pitfalls of using LLMs for tasks such as data processing, report generation, and other supportive roles, while building a nuanced understanding of potential errors and biases. Given the “generative” feature of LLMs, guardrails will need to be placed on these types of integrations to assure both providers and patients alike that their use is safe.

Laboratory information system (LIS) managers and pathology informaticians deeply involved in LIS use must be equipped with the knowledge to integrate LLMs into existing LIS and middleware solutions. Further, strategies will need to be developed to account for effective data management, security, validation, and verification of LLMs when use in both operational and clinical use within the labs. Administrators should be educated on the potential benefits, costs, and risks associated with LLM implementation, enabling them to make informed decisions on resource allocation and strategic planning.

Investments in infrastructure will almost certainly be required. For organizations that intend to develop or fine-tune models locally, this would likely include facilities for secure storage and processing capabilities to handle large volumes of data. A significant amount of manual effort would typically be necessary to prepare and curate a clean, unbiased data set for fine-tuning LLMs. Luckily, the infrastructure requirements for hosting already trained models are more modest. Each organization must choose a strategy that aligns with local capabilities and approaches to risk management. For example, an organization with pre-existing informatics expertise and infrastructure and a low tolerance for sharing data with third parties would be more likely to consider self-hosting LLMs (to the extent that they are available), whereas one without local infrastructure but with the ability to establish business relationships with vendors that host sensitive data might be more open to the use of external services.

Once an infrastructure strategy is defined, the next consideration is how much your institution is willing to pay and for how long. As an example, ChatGPT costs *$700k per day*^39^ to operate. The cost to train GPT4 was about $63M^40^—not counting all the technical staff needed to do the training, and data curators to organize and clean it, and years of preparation. Each of the dozens of individuals required for this type of undertaking demand well over 6 figure salaries. In other words—it is not cheap in terms of time or money, and de-novo generation of large-scale LLMs is likely to remain beyond the capabilities of healthcare organizations for the foreseeable future. It is important for organizations to recognize their scientific, engineering, data quality, and financial limitations before investing millions into an AI program that will never be possible with their current resources. Luckily, the infrastructure requirements for hosting already trained models are more modest—with OpenAI’s models costing on the order of 10–20 cents per 1000 tokens,^41^ has no upfront infrastructure build, and leverages web-based technology that should be familiar to hospital IT professionals.

In contrast to their relative deficit of raw computing power, large healthcare organizations may have access to critical data that technology-focused companies lack. Ensuring that any data that is available and appropriate for use is FAIR (Findable, Accessible, Interoperable, Reusable)^42^ can greatly enhance the efficiency and effectiveness of LLMs in processing and analyzing information. This requires standardizing data formats, adopting metadata standards, and using open, non-proprietary formats.

It is currently impossible to predict which models, products, or modes of operation will become dominant in healthcare. However, EHR vendors have already begun to incorporate LLM capabilities into their products, and LIS products are likely to follow suit. For instance, a major EHR vendor has partnered with Microsoft and OpenAI to develop AI-powered analytics capabilities in its business analytics product, with pilot projects at several major academic centers.^43^, ^44^, ^45^

However, organizations with the necessary capabilities may choose to develop and deploy their own models. Any non-integrated solution will require the development of Application Programming Interfaces (APIs) to exchange data between systems, so organizations must plan to work with EHR and LIS vendors to develop these capabilities.

The implementation of AI tools also requires the establishment of robust institutional governance structures capable of addressing compliance and legal liability issues efficiently. Healthcare organizations without such structures may face substantial difficulties when introducing LLMs. The development of clear and well-communicated policies is essential for guiding responsible and suitable use of LLMs within organizations.

Formulating these policies will likely involve intensive dialog and consensus-building, possibly even at the national level. Involving government agencies like the National Institute of Standards and Technology, Centers for Medicare & Medicaid Services, and the Food and Drug Administration (FDA) in these conversations can be beneficial, especially since the implications of clinical AI deployment in healthcare are vast, reaching beyond the individual organizations deploying LLMs.

Professionals in compliance and risk management will also need to be thoroughly acquainted with the legal, ethical, and regulatory aspects of utilizing LLMs in healthcare settings. They play a vital role in ensuring LLM applications adhere to privacy, security, and compliance standards, and in addressing potential risks linked with their use. Integrating LLMs into existing systems and understanding the regulatory implications of this process is an ongoing, potentially expensive endeavor.

As these technologies evolve, new tools such as NeMo Guardrails^46^ may enable organizations to translate policies into technical boundaries for internally hosted LLMs. This and similar tools might become instrumental for deploying AI instances with access to sensitive PHI, financial, or information that could be purposefully or inadvertently convinced to reveal it because of a query. Nevertheless, it is important to understand that optimized versions of LLMs and similar software will eventually run locally (even on mobile devices and phones), without any associated technical institutional restrictions. For those situations, the traditional data leakage protection tools and access controls will need to evolve to protect the organization’s data.

To fully capitalize on the potential of LLMs, all stakeholders must develop an understanding of what is (and is not) possible with these models, which will enable them to ask new questions and explore innovative applications (see Table 2). Security and privacy must be at the forefront of LLM education and training, ensuring that new users are aware of the importance of protecting sensitive data and maintaining compliance with relevant regulations. By fostering a culture of continuous learning and responsible LLM usage, healthcare organizations can maximize the benefits of this transformative technology in pathology informatics.

**Table 2:** Preparing individuals and organizations for life with LLMs.

As an individual	As an organization
LLMs are already useful as productivity tools to support day to day activities. Make a conscious effort to experiment with LLMs while performing common tasks (as appropriate) to gain familiarity with uses and limitations ● Consumer-facing applications provided by major technology companies have the lowest barrier to entry. Become familiar with freely available services such as ChatGPT (https://openai.com/blog/chatgpt), Bing Search (https://www.bing.com/), or Google Bard (https://bard.google.com/). ● Enroll in early access programs for these and other services as they become available. For example, at the time of writing, ChatGPT Plugins (https://openai.com/blog/chatgpt-plugins) offers a waiting list for access, as does Google Search Labs (https://blog.google/products/search/search-labs-ai-announcement-/) for previewing AI integration into its search and office products. ● Expanded capabilities (e.g., faster response times and access to more powerful models) can be provided via a subscription, for example to ChatGPT Plus (https://openai.com/blog/chatgpt-plus). ● More technical users can obtain access to APIs for integration with editors and programming tools (https://openai.com/blog/openai-api). Early access programs for exploration of features in development are available for API services as well. ● Those with experience in data science (particularly in the Python ecosystem) should consider experimenting with open-source models (e.g., https://huggingface.co/models) to gain familiarity with concepts such as fine tuning, embeddings, tokenization, etc.	Consider the entire spectrum of use cases and define strategies for lowering barriers to entry at each level of risk and complexity. ● Establish baseline expectations for the appropriate use of internal and external resources and be prepared to iterate on the resulting policy as new resources become available; recognize that the process of developing such a policy itself provides value through the identification and engagement of stakeholders. Start with a narrow scope (“Can I use my personal ChatGPT account at work to summarize email?”) to address immediate use cases. Critically, inform leaders and compliance officers of the considerations for balancing risk and opportunity. ● Identify “superusers” with the skills and willingness to share insights and use cases within the organization. ● Provide access to LLM-based applications (either through commercial subscriptions or self-hosted infrastructure) to lower barriers for use and adoption, particularly for trainees and technical staff. ● For academic institutions, establish research collaborations with investigators with expertise in NLP; engage with the institutional IRB as early as possible. ● Any organization with objectives that include local development of applications for any operational, research, or clinical use case should ensure that they have local expertise in open source application hosting and development to extend opportunities beyond those offered by vendors (see https://simonwillison.net/2023/May/4/no-moat/ for a perspective on the likelihood that open source models will meet or exceed the capabilities of commercial products).

## Laws and regulations

Regulatory challenges are increasingly emerging for the development and deployment of LLMs in healthcare, particularly for commercial software. In April 2023, the FDA issued a draft guidance recommending the submission of a “predetermined change control plan” for AI-enabled healthcare software.^47^ This plan would permit software developers to make iterative, non-significant changes to the AI model, provided they include a description of the modifications, a modification protocol, and an impact assessment. However, given the intricate nature of LLMs, describing modifications or assessing their impact on the model may prove challenging.

LLMs that interface with clinicians, especially those that provide patient-care guidelines or influence clinical decision-making, may be classified as “clinical decision support” (CDS) software, necessitating additional regulatory scrutiny.^48^ CDS software that directly enhances or guides clinical judgement, particularly in time-sensitive situations, or does not allow clinicians to independently review the basis of the software's recommendations, may be regulated by the FDA as “software as a medical device”. This classification is similar to in-vitro diagnostic tools such as laboratory test devices.^49^ The Office of the National Coordinator for Health Information Technology has also proposed guidelines for how health IT developers should develop and certify CDS software, including “predictive decision support interventions”.^50^

While these rules will apply to commercial software sold specifically for healthcare purposes, it remains unclear to what extent they may also apply to in-house LLM solutions, particularly those that incorporate and modify existing commercial software.^51^ As new technologies continue to challenge traditional and static definitions, such as “medical device” and even “modification”, the regulatory landscape is certain to evolve.

## Unresolved challenges

### Explainability, interpretability, and performance assessment

As the integration of LLMs into pathology informatics expands, several challenges must be navigated to ensure their responsible and effective use. The first of which is how to optimally integrate human expertise with the outputs generated by LLMs. This remains an open area of exploration throughout all fields of machine learning, but is particularly difficult in LLM applications, as the assessment of performance is often non-descript or subjective. It should not be assumed that “human-in-the-loop”, nor fully autonomous applications will be superior for all applications.^52^, ^53^, ^54^, ^55^ Experimental results have been mixed in this regard. For the use-case of detecting wrong-blood-in-tube errors, the integration of human and model predictions actually led to poorer performance than the model alone,^54^ while radiologists also displayed significant automation neglect and poorer accuracy when review cases that had been previously labeled by an AI.^55^ We recommend a case-by-case assessment with a thorough, pre-defined primary endpoint for comparing autonomous versus human-in-the-loop implementations until a more universal framework is discovered. Establishing validation procedures for LLMs will be particularly challenging due to the inherently unpredictable nature of these models. LLMs, by their design, can generate a wide array of outputs even with identical inputs. This makes conventional validation mechanisms, often used for deterministic systems, less effective. The most effective “validation” method for LLM use involves the integration of human oversight and the creation of tools that can sufficiently constrain the model's output.

One crucial feature of a model that is being deployed in a human-in-the-loop framework is explainability. In machine learning, explainability refers to the ability to attribute the value to each feature to its effect on the final output. While approaches like SHAP^56^ and LIME^57^ have provided useful tools for other facets of machine learning, explainability techniques for LLMs are very much in their infancy, and have practically been limited asking the models for a detailed, step-by-step explanation of which it produced the output that it did.^58^ This is far cry from a truly explainable system that can be used to troubleshoot incorrect responses or motivate further exploration into correct ones.

Interpretability in machine learning refers to ability to look “under the hood” of the model to uncover insights into how a model comes to its conclusions. While similar to explainability, an apt analogy is that a car is explainable for most drivers (gas pedal causes acceleration, brake pedal causes deceleration, steering wheel turns the car), however, the inner workings of the car’s combustion engine is only interpretable to highly specialized engineers and mechanics. The longstanding criticism of neural network approaches as uninterpretable “black boxes” remains relevant to LLMs, as there is no meaningful manner for humans to make sense of the complex activation patterns of nodes and layers within the models.

The lack of explainability and interpretability is particularly concerning when LLMs produce demonstrably incorrect output or “hallucinations,” as there is no way to trace the error to its root cause or make immediate adjustments. Although some research has been conducted to enable interpretable neural networks, more progress is needed to enable both clear interpretations of the processes and enduring, rapid corrective measures.^59^

Inextricably linked with explainability is the issue of performance assessment. The often subjective nature of LLM outputs renders typical methods such as sensitivity and specificity inapplicable. The development of suitable and scalable validation strategies for LLMs is a significant challenge and represents a crucial frontier in the journey towards adoption. Validation strategies could range from comparing LLM outputs against manually curated gold-standards or running controlled experiments with known outcomes, to real-time monitoring and feedback systems for quality control. However, these strategies should be designed with the end users in mind and should address real-world complexities and variations. Ensuring that the truth sets used for validation are as accurate and reliable as possible is vital. This may require the development of new methodologies, consensus-building among experts, or leveraging other advanced technologies to establish more robust ground truths.

The return on investment (ROI) for LLMs in pathology informatics is not immediately apparent, with initial ROI focusing on improved quality, operational efficiencies, and cost savings. Organizations need to carefully evaluate potential use cases and success criteria prior to investment. By prioritizing those applications that offer the greatest tangible and measurable value, such as improving diagnostic accuracy, streamlining workflows, or reducing manual processes, organizations can build a compelling case for investing in LLM technology.

### Algorithmic fairness and bias mitigation

The early adoption of machine learning models at enterprise scale has led to some tremendous, high-profile examples in which a lax approach to the assessment of algorithmic fairness prior to deployment has contributed to inequity through its predictions. Algorithms trained on insurance claims data allocate substantially fewer resources to Black patients,^60^ law enforcement applications have had disastrous unintended consequences,^61^ the Apple Card approved men for larger lines of credit than their otherwise identical-on-paper wives, and many, many more.

Increased adoption of AI in healthcare has the potential to lead to more objective decisions, but making decisions free from the biases of human decision-making is more complex. AI algorithms are developed using data generated in a society that is not free of bias.^39^ As a result, bias can be incorporated into AI at each step in the algorithm development pipeline. Indeed, numerous examples exist where the use of algorithms in healthcare has led to biased outcomes that negatively impact marginalized populations. These include underdiagnosis of abnormal chest radiographs in underserved populations,^40^ decreased classification performance in identifying pigmented skin lesions in dermatological images from individuals with skin of color,^41^ and unequal allocation of healthcare resources for Black patients,^42^ among others.

LLMs are trained on real-world data that may be imbued with societal biases, making them susceptible to incorporating and propagating these biases. Previous studies have demonstrated that LLMs may capture stereotyped beliefs represented in training sets.^43^, ^44^, ^45^ There is a risk that LLMs trained on healthcare data may incorporate similar, or perhaps even worse, biases, particularly against marginalized groups. These groups may be disproportionately underrepresented in training data, as they are historically underrepresented in healthcare and research datasets^46^ and are more prone to having missing data.^47^ Additionally, provider-level biases, such as disparate treatment due to unconscious biases^48^ or negative sentiments present in the clinical notes of underrepresented patients, may also contribute to the bias present in the datasets used to train LLMs.

While it is impossible to have a truly unbiased algorithm in a biased society, steps can be taken to reduce the propagation of societal biases through LLMs.^49^ For example, strategies such as up- and down-sampling may reduce underrepresentation in training datasets.^50^ Developers can also incorporate a “fairness term” into the objective functions used to train LLMs to penalize biased outputs, even if it comes at the expense of overall accuracy.^51^^,^^52^ Finally, processes should be implemented to evaluate and correct for bias in the output of LLMs post-deployment.^51^ Ultimately, organizations will need to adopt a proactive and intentional approach to ensure that any LLMs deployed in practice do not exacerbate disparities for underrepresented or marginalized populations.

### Safety, security, and social responsibility

Given the swift progress in LLM research and development, continuing education is essential for keeping healthcare professionals abreast of the latest advancements and best practices. This includes revisiting and updating training programs, guidelines, and policies as the technology matures. The rapid pace of advancements in LLM technology often outpaces the publication cycle of traditional scientific literature. As a result, healthcare professionals may need to seek out alternative channels, such as preprint servers, blogs, general news outlets, and social media, to stay updated on the latest developments.

The open letter titled “Pause Giant AI Experiments” urges the AI research community, companies, and governments to pause the deployment of large-scale AI systems, such as LLMs, and to consider the potential consequences of their use.^62^ The letter highlights the potential risks associated with LLMs, including biases, misinformation, and negative environmental impacts. It calls for comprehensive risk assessments, public input, and the establishment of regulatory frameworks to ensure the responsible development and deployment of AI technologies. Additionally, the letter emphasizes the need for transparency and collaboration among stakeholders to address the challenges and to harness the benefits of AI advancements for society. The pathology informatics community has a small, but essential role to play as LLM technology advances, and should be aware of issues the larger AI community is facing.

While efforts to constrain responses are ongoing,^63^ LLMs remain vulnerable to prompt injection attacks, which can be used for malicious data exfiltration, modification of subsequent behavior, or issuing rogue commands to systems interfaced with them via APIs. Therefore, secure system architecture, access permissions, timely updates, and monitored logging should be integral parts of the system design. A comprehensive security approach should also include threat modeling, risk assessments, and user education. Even commercial entities with experts in data security and protection intellectual property have already experienced code leaks,^64^ demonstrating the challenges in controlling the development of such models.

### Technical and operational considerations

The context window, or “prompt size”, of large language models is limited and varies depending on the specific model. For instance, GPT-3's limit is 4096 tokens, encompassing both prompts and responses, while variants of GPT-4 range from 8192 to 32 768 tokens. Given that approximately 4 tokens equate to 3 words, it may not currently be feasible to include an entire textbook or a patient's complete chart in the prompt, followed by a question, or to request an LLM to compose an extensive publication. The addition of API interfaces to external systems may also help address this issue, but future advancements, such as expanding the context window, are anticipated to provide more effective solutions. For example, the MPT-7B LLM, using the “Attention with Linear Biases” (ALiBi) method,^65^ can already accept more than 60 000 tokens, and the Claude 2 chatbot pushes its limit to 100 000 tokens.^66^

Initiating a project to fine-tune an existing LLM for a specific task can require significant resources. These include the extraction, curation, and preparation of suitable training data, the availability of experienced personnel, and access to appropriate hardware, either via a cloud service or locally, to facilitate training and validation iterations. While current implementation solutions are limited to engineering alternative prompts, fine-tuning, or retraining, recent advances in end-to-end frameworks such as LangChain (cite LangChain github) have reduced the barrier to more state-of-the-art approaches such as retrieval augmented generation (cite Lewis). Additional resources may be needed for integration with data sources via APIs and tools such as Sidekick.^67^ Given the rapid pace of progress in this field, the team must be prepared to adopt and refine the latest, advanced LLMs to achieve better accuracy and higher quality responses.

Lastly, organizations must adopt an agile approach to decision-making. AI technologies are emerging rapidly, and the first-mover advantage will belong to those who can swiftly address the spectrum of technical, legal, regulatory, and ethical barriers as they arise. There is no “one size fits all” method to address these challenges. Interdisciplinary teams representing all stakeholders will be required to navigate the complex interplay of these different barriers. However, these teams should be formed with the goal of making authoritative decisions when needed. Including AI development in organizational strategic planning, such as budgeting appropriate resources, acquiring the required expertise, and prioritizing the implementation of advancements and upgrades, would likely facilitate responding to the newest technological capabilities.

Each of the topics discussed above will require the establishment of governance with participation from stakeholders who bring the necessary skill sets to the table. For example, technically proficient organizations that fail to arrive at an internal consensus on how to address ethical concerns will be left behind along with organizations that can streamline approvals related to data security but lack the necessary infrastructure. The lack of a decision on how to adopt these technologies is a decision not to use them.

## Conclusions

In conclusion, this paper has explored the transformative potential of Large Language Models (LLMs) in the field of pathology informatics. It has highlighted the diverse applications of LLMs, from data discretization to generating differential diagnoses and summarizing case visits. However, it has also emphasized the need for careful and responsible use of these models, considering factors such as independent verification, risk of omission, and potential biases. The paper underscored the necessity of significant investment in infrastructure, data management, and security, as well as the importance of comprehensive education and training for various stakeholders. It also discussed the challenges that lie ahead, including the risk of overreliance on LLMs, the need to keep pace with rapid technological advancements, and the development of suitable validation strategies. As we move forward, the judicious and informed use of LLMs can unlock their full potential in pathology informatics, leading to improved patient care and a new era of efficiency in healthcare. However, this journey will require continuous learning, adaptation, and a commitment to addressing the technical, legal, regulatory, and ethical barriers that emerge along the way.

## Funding

This research did not receive any specific grant from funding agencies in the public, commercial, or not-for-profit sectors.

## Statement

During the preparation of this work the authors used ChatGPT (GPT4—May 24, 2023 version) in order to improve readability and flow. After using this tool/service, the authors reviewed and edited the content as needed and take full responsibility for the content of the publication.

## Declaration of Competing Interest

The authors declare that they have no known competing financial interests or personal relationships that could have appeared to influence the work reported in this paper.
